# Combining distance and anatomical information for deep-learning based dose distribution predictions for nasopharyngeal cancer radiotherapy planning

**DOI:** 10.3389/fonc.2023.1041769

**Published:** 2023-02-28

**Authors:** Xinyuan Chen, Ji Zhu, Bining Yang, Deqi Chen, Kuo Men, Jianrong Dai

**Affiliations:** ^1^ National Cancer Center/National Clinical Research Center for Cancer/Cancer Hospital, Chinese Academy of Medical Sciences and Peking Union Medical College, Beijing, China; ^2^ National Cancer Center/National Clinical Research Center for Cancer/Hebei Cancer Hospital, Chinese Academy of Medical Sciences, Langfang, China

**Keywords:** dose-prediction, minimum distance, anatomical information, deep-learning, radiotherapy treatment planning

## Abstract

**Purpose:**

Deep-learning effectively predicts dose distributions in knowledge-based radiotherapy planning. Using anatomical information that includes a structure map and computed tomography (CT) data as input has been proven to work well. The minimum distance from each voxel in normal structures to planning target volume (DPTV) closely affects each voxel’s dose. In this study, we combined DPTV and anatomical information as input for a deep-learning–based dose-prediction network to improve performance.

**Materials and methods:**

One hundred patients who underwent volumetric-modulated arc therapy for nasopharyngeal cancer were selected in this study. The prediction model based on a residual network had DPTV maps, structure maps, and CT as inputs and the corresponding dose distribution maps as outputs. The performances of the combined distance and anatomical information (COM) model and the traditional anatomical (ANAT) model with two-channel inputs (structure maps and CT) were compared. A 10-fold cross validation was performed to separately train and test the COM and ANAT models. The voxel-based mean error (ME), mean absolute error (MAE), dosimetric parameters, and dice similarity coefficient (DSC) of isodose volumes were used for modeling evaluation.

**Results:**

The mean MAE of the body volume of the COM model were 4.89 ± 1.35%, highly significantly lower than those for the ANAT model of 5.07 ± 1.37% (*p<*0.001). The ME values of the body for the 2-type models were similar (*p >*0.05). The mean DSC values of the isodose volumes in the range of 60 Gy were all better in the COM model (*p*<0.05), and there were highly significant differences between 10 Gy and 55 Gy (*p*<0.001). For most organs at risk, the ME, MAE, and dosimetric parameters predicted by both models were concurrent with the ground truth values except the MAE values of the pituitary and optic chiasm in the ANAT model and the average mean dose of the right parotid in the ANAT model.

**Conclusions:**

The COM model outperformed the ANAT model and could improve automated planning with statistically highly significant differences.

## 1 Introduction

Inverse treatment planning is an important step for modern radiotherapy, such as intensity-modulated radiotherapy (IMRT) and volumetric-modulated arc therapy (VMAT). Dose and dose-volume–based objectives/constraints are essential in most commercial inverse planning systems. The optimal objectives/constraints for different patients, even if diagnosed as cancers of the same stage and grade, are very different due to variations in the morphology and position of the tumor and normal tissues. The process of finding personalized optimal objectives/constraints is time consuming and depends on the experience of the planner (1–4).

Some knowledge-based radiotherapy planning (KBRP) methods have been developed to substitute the human-dominated treatment planning process in recent decades (5–7).One of the key steps of KBRP approaches was to predict three-dimensional (3D) dose distributions for setting optimal objectives/constraints (8, 9). In early studies, the geometrical features of the organs at risk (OARs) and target(s) were manually extracted for the modeling of dose-prediction with machine learning methods (10). KBRP methods have been proven to have good performance in sparing OARs, decreasing inter-operator differences, and improving planning efficiency. The extracted geometric features of KBRP include the minimum distance to target and OARs, as well as angles related parameters (11, 12). Among them, the minimum distance to target has been widely used as an effective handcraft geometric feature in dose prediction modeling with machine learning which can capture the general slope of dose gradient outside PTV with lower average dose as the distance increases (13–15).

More recently, some research groups have worked on deep-learning methods to predict patient-specific dose distributions automatically. As one of the solutions of deep-learning methods, convolutional neural networks (CNNs) show potential to predict dose distribution (16, 17). Some research groups have explored deep-learning-based dose-prediction methods that use anatomical inputs combined with computed tomography (CT), OARs, and targets (18–20). The normalized mean square error and dose difference for rectal cancer radiotherapy plans were 0.001 and 0.4%. Ma et al. (21) introduced a deep-learning method for dose-prediction using inputs of contours (PTV and OARs) and dosimetric features. Compared with the traditional contours-based model, the model including dosimetric features could significantly improve the dose prediction accuracy for target with p< 0.001. Our research group also developed a CNN with 101 layers that used the inputs of OARs anatomical information, targets, and out-of-field distance to predict dose distributions which could improve the mean absolute dose from 5.5% to 4.7% (16).

Although deep-learning methods with anatomical information perform well on dose-prediction tasks, the geometric features are also related to the dose distribution. Yue et al. (22) applied the distance information to guided deep-learning including signed boundary distance map for regions of interest (ROIs) and the Euclidean distances from all body voxels to the center of the CTV. Different from that method, we introduced the minimum distance from each voxel in normal structures to planning target volume (DPTV) which was more common used for machine learning based KBRT. It is directly related to features for dosimetric parameter prediction and may also be an important parameter for training deep-learning models. The study aim was to propose a method that combines distance and anatomical information (structure maps and CT) as input for deep-learning based dose-prediction network training to improve model performance.

## 2 Materials and methods

### 2.1 Patient data

One hundred patients with nasopharyngeal cancer who received VMAT between 2016 and 2020 were enrolled in this study. Thermoplastic masks (Klarity Medical, Guangzhou, China) were used to immobilize the patients in the supine position. The simulation CT images were acquired on a Somatom Definition AS 40 (Siemens Healthcare, Forchheim, Germany) or a Brilliance CT Big Bore (Philips Healthcare, Best, The Netherlands) system with the same settings of a 3 mm slice thickness and a 512 × 512 matrix.

A 3 mm margin was applied around the gross tumor volume of the nasopharynx (GTVnx) and clinical target volume to create the planning GTVnx (PGTVnx) and PTV, respectively. The metastatic lymph nodes of gross tumor (GTVnd) were also contoured.

The combination of PGTVnx and GTVnd was named “Boost”. The prescription of PGTVnx and GTVnd was 69.96 Gy in 33 fractions (2.12 Gy/fraction). The prescription of the PTV was 60.06 Gy in 33 fractions (1.82 Gy/fraction). Twenty-one OARs were adopted for inverse treatment planning constraints, including larynx, biliteral lens, biliteral mandible, optic chiasm, biliteral optic nerves, biliteral parotids, pituitary, biliteral temporomandibular joints (TMJs), biliteral temporal lobes, thyroid gland, trachea, brain stem and the corresponding planning organ at risk volume (PRV) with 3mm margin, spinal cord, and PRV with 5mm margin.

The VMAT plans were optimized in the Pinnacle 9.10 version treatment planning system (TPS) (Philips Radiation Oncology Systems, Fitchburg, WI, USA) with 6 MV photons. Two opposite coplanar dynamic full arcs, each consisting of 91 control points, were used to generate all plans. The dosimetric objectives of the target volumes and OARs for direct optimization of machine parameters were consistent. The final dose grid resolution was set as 0.4 × 0.4 cm in the TPS and the dose maps were interpolated into the same pixel size with the corresponding CT image. The delineations of the regions of interest (ROIs) and treatment plans were reviewed carefully and approved by our radiotherapy team that included senior radiation oncologists and senior physicists.

A 10-fold cross validation was conducted for 100 cases that were randomly partitioned into 10 equal-sized subsets. The model was trained using nine subsets (90% of the data) and tested using the remaining subset (10% of the data). Ten models were trained using the same procedure, and 10 sets of results were obtained to evaluate the performance of each established deep-learning method. The dose distribution maps of the approved plans were adopted as ground truth (GT) for model estimation.

### 2.2 Generating the DPTV maps and structure maps

To incorporate the distance information for deep-learning methods, the DPTV maps were generated as inputs. The DPTV is defined as the minimum distance to PTV surface for each voxel of normal tissue that was outside the PTV but within body range. The DPTV was calculated in 3D to search the proximity distance to PTV according to equation 1.


Eq. 1 $${\text{DPTV}}_{i}=\text{min }\left\{Distance\left(Voxel_{i},\;Voxel_{PTV}\;\right)\right\}\;$$

$$Voxel_{i}\in Body\;\cap^{}\;Voxel_{i}\notin PTV,\;\;Voxel_{PTV}\in PTV$$

For each slice of the DPTV map, the PTV voxels were labeled “0” and DPTV*_i_* was the label value of each corresponding *Voxel_i_*. The voxels in the outside body range were labeled “0.”

The same method based on our previous proposed study was used to generate the structure maps (16). The targets, OARs, body, and out-of-field voxels were assigned separate unique labels. The overlap of the OARs and target was labeled with their summation. The out-of-field voxels were also specified because they have been proven to achieve more accurate dose-prediction.

### 2.3 Experiments

#### 2.3.1 Input and output of the networks

To identify the better combination of inputs for dose-prediction models, two kinds of inputs were compared: The first one was to use anatomical maps as two-channel inputs that included structure maps and CT to train a deep-learning anatomical (ANAT) model. The second one was to use combined anatomical maps as three-channel inputs that included DPTV maps, structure maps, and CT to train a deep-learning combined (COM) model. The outputs were the corresponding dose distribution maps.

#### 2.3.2 Architecture of the deep-learning networks

**Figure 1:**
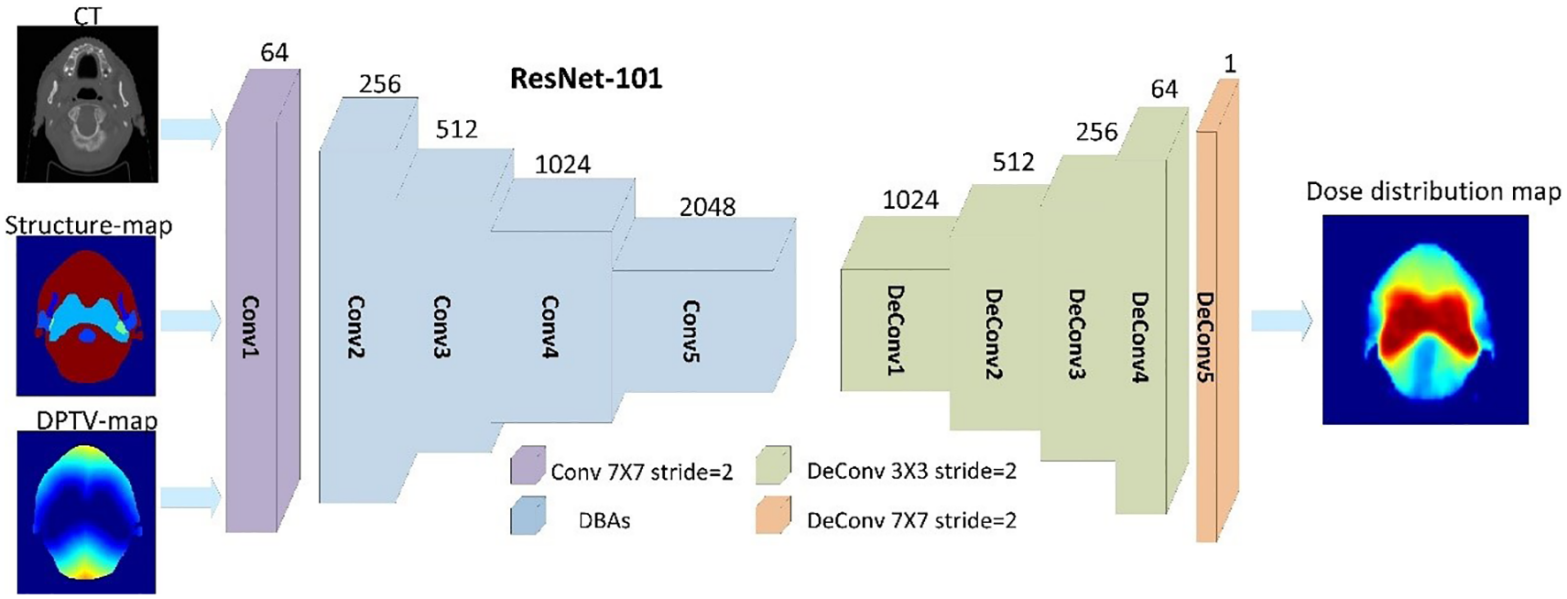
Training workflow based on 101 with three input channels (CT, structure maps, and DPTV maps) of the COM model.

The overall end-to-end workflow of CNNs was proposed to predict pixel-wise dose distributions. The ANAT model and COM model were trained separately with the same network architecture. The training workflow of the COM model is shown in **Figure 1**. The main generator of the network was based on Resnet with 101 layers, which had been introduced in our previous study (16). Conv1 is a 7×7 convolutional layer with 64 filters. A max-pooling operation is then performed for downsampling. Conv2, Conv3, Conv4, and Conv5 consist of 3, 4, 23, and 5 deeper bottleneck architectures (DBAs), respectively (23). Each DBA has three convolutional layers of 1×1, 3×3, and 1×1 and a connection. The output of Conv5 is 1/8 of the original image. An upsampling based on the fractionally-strided deconvolution is used to restore the image resolution. PyTorch was used to implement the process of model training and testing on a workstation equipped with an Intel^®^ Core i7 CPU (3.4 GHz) and a TITAN XP graphics card. A total of 100 epochs were set for training to ensure loss convergence.

#### 2.3.3 Quantitative evaluation

##### 2.3.3.1 Voxel-based comparison

The accuracy of the predicted dose distributions by the two models were evaluated against the corresponding GT dose voxel-by-voxel. The voxel-based mean error (ME) and mean absolute error (MAE) were used as the evaluation indexes for outlines of body (24). For each patient, the ME and MAE values in the range of the body, whole normal tissue, and each ROI were separately calculated, according to equation 2 and 3:


Eq. 2 $$ME=\;\frac{1}{N}\frac{\sum_{j=1}^{N}\frac{1}{M}\sum_{i=1}^{M}\left(D_{Pred}\left(i\right)-D_{GT}\left(i\right)\right)}{prescription\;dose}*100\%\;$$

Eq. 3 $$MAE=\;\frac{1}{N}\frac{\sum_{j=1}^{N}\frac{1}{M}\sum_{i=1}^{M}\left|D_{Pred}\left(i\right)-D_{GT}\left(i\right)\right|}{prescription\;dose}*100\%\;$$

where *i* is the index of the voxel in each ROI for each patient and *M* is the total number of the involved voxels. *D_Pred_* (*i*) and *D_GT_* (*i*) are the predicted and GT dose of a voxel *i*, respectively, *j* is the index of the case, and *N* is the total number of cases in the test set.

##### 2.3.3.2 Dosimetric comparison

Some critical dosimetric parameters of OARs related to the setting of inverse optimization, including the mean dose and D5% (doses delivered to 5% volume of an OAR), were calculated for both models.

##### 2.3.3.3 DSC of isodose volumes comparison

The performance of different models were also evaluated by the 3D DSC (25) of isodose volumes calculated as equation 4.


Eq. 4 $$DSC_{i}=\;\frac{2\left(IV_{iPred}\cap^{}IV_{iGT}\right)}{IV_{iPred}+IV_{iGT}}$$

where *DSC_i_* describes the degree of agreement between the predicted isodose volume in dose *i* (*IV_iPred_*) and the corresponding isodose volumes of GT(*IV_iGT_*). The *DSC_i_* of continual dose *i* within the range of prescription dose (1 Gy - 60 Gy) was calculated respectively for each model and plotted for comparison. Furthermore, the values of *DSC_i_* of dose *i* from 5 Gy to 55 Gy with a 5 Gy bin were extracted for statistical analysis.

##### 2.3.3.4 Statistical analyses

Differences in the values of MAE and DSC between the two models were assessed by performing the paired *t*-test. The critical dosimetric parameters were compared against GT values and evaluated by the paired *t*-test. All of the statistical analyses were performed in IBM SPSS Statistics for Windows software (version 25.0; IBM Corp., Armonk, New York, USA). All of t-tests were two-sided. P values<0.05 were considered to be indicative of statistical significance.

## 3 Results

**Figure 2:**
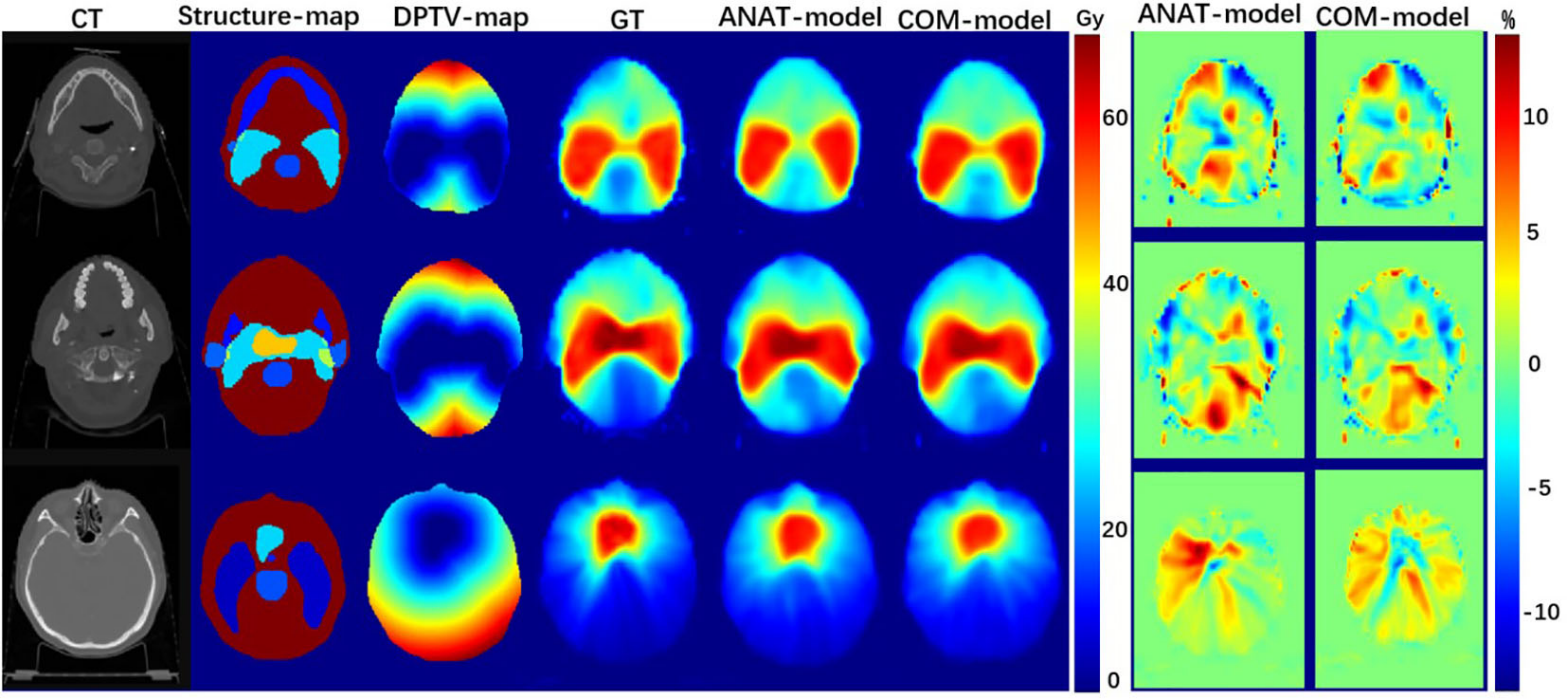
Examples of dose maps predicted by the COM model and ANAT model: the first column, the CT maps as input; the second column, the structure-maps as input; the third column, the DPTV-maps as input; the fourth column, the dose maps of approved treatment plans; the fifth column, the dose maps predicted by ANAT-model; the sixth column, the dose maps predicted by COM-model; the seventh column, the dose-difference maps for ANAT-model; the eighth column, the dose-difference maps for COM-model.

**Figure 3:**
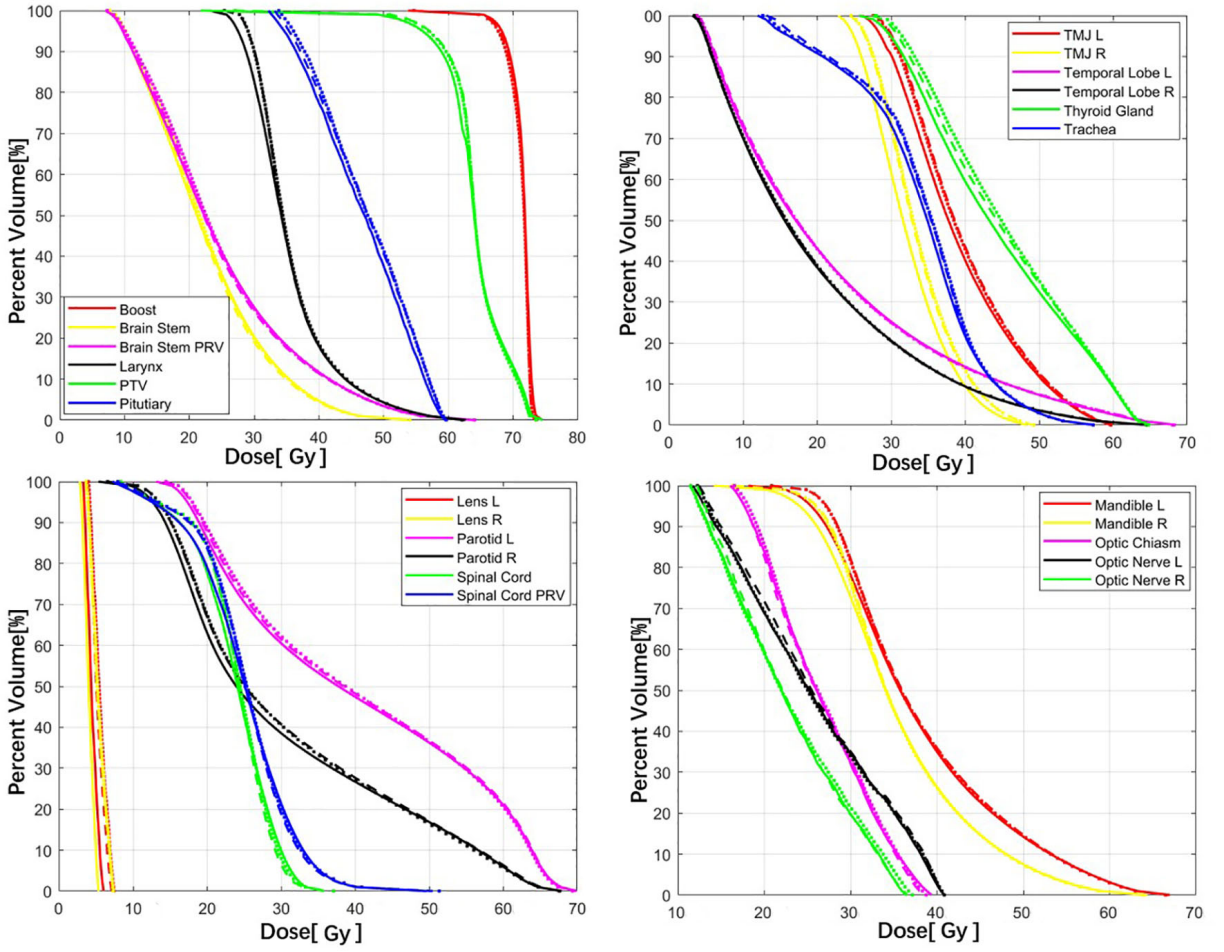
Comparison of the mean DVHs between the COM model (dash lines), ANAT model (dot lines), and GT (solid lines).

As shown in **Figure 2**, both the COM model and ANAT model predicted similar dose maps in acceptable agreement with the GT for visual comparison. **Figure 3** illustrates the mean DVHs of all patients in the test dataset. The DVH curves predicted by the COM model and ANAT model were close to those of the clinically approved GT for most of the OARs and targets.

The MAE of the body volume was lower for the COM model (4.89 ± 1.35%) compared to the ANAT model (5.07 ± 1.37%) (*p*<0.001). As **Table 1** shows, comparing the MAEs for most OARs, there were no significant differences between the two models. The MAE was significantly lower in the COM model than the ANAT model only for the pituitary and optic chiasm. The mean MAEs of the 21 OARs were 5.45 ± 1.80% for the ANAT model and 5.21 ± 1.49% for the COM model. For whole normal tissue, the mean MAE was 5.18 ± 1.22% of the COM model, which was also highly significantly lower than the 5.39 ± 1.25% of the ANAT model (*p*<0.001).

**Table 1:** Comparison of the MAE for each OAR and target.

OARs	ANAT model (%)	COM model (%)	OARs and targets	ANAT model (%)	COM model (%)
BrainStem PRV	5.68 ± 1.74	5.63 ± 1.83	Pituitary*	9.07 ± 6.08	7.63 ± 5.41
BrainStem	5.66 ± 1.90	5.66 ± 2.00	SpinalCord PRV	6.00 ± 1.99	6.05 ± 1.99
Larynx	4.35 ± 1.52	4.25 ± 1.39	SpinalCord	6.19 ± 2.26	6.17 ± 2.28
Lens L	1.99 ± 1.71	2.13 ± 1.93	TMJ L	4.77 ± 2.12	4.72 ± 2.50
Lens R	1.85 ± 1.41	2.00 ± 0.67	TMJ R	5.31 ± 2.82	5.50 ± 2.99
Mandible L	4.77 ± 1.20	4.60 ± 1.14	Temporal Lobe L	5.03 ± 1.70	4.75 ± 1.89
Mandible R	5.11 ± 1.49	5.03 ± 1.57	Temporal Lobe R	4.71 ± 1.68	4.52 ± 1.62
Optic Chiasm*	9.22 ± 5.17	8.08 ± 4.62	Thyroid Gland	5.01 ± 2.29	4.73 ± 1.48
Optic Nerve L	7.66 ± 4.24	7.09 ± 4.87	Trachea	4.48 ± 1.24	4.31 ± 1.21
Optic Nerve R	7.00 ± 4.57	6.41 ± 4.19	Parotid R	5.66 ± 2.38	5.54 ± 1.93
Parotid L	4.90 ± 1.54	4.80 ± 1.54	PTV	2.54± 2.44	2.52 ± 2.42
Boost	1.34 ± 0.30	1.30 ± 0.41			

*p<0.05.

The ME values of the body for the COM and ANAT models were similar (*p* = 0.575) and were 0.21 ± 1.85% and 0.18 ± 1.91%, respectively. As shown in **Table 2**, the mean ME values of 21 OARs and two targets were also very similar but with no statistically significant differences (*p >0.05*). For whole normal tissue, the mean ME of the COM model was 0.20 ± 1.80%, which was very similar to the ANAT model with 0.14 ± 1.89% of (*p* = 0.412).

**Table 2:** Comparison of the ME for each OAR and target.

OARs	ANAT model (%)	COM model (%)	OARs and targets	ANAT model (%)	COM model (%)
BrainStem PRV	-0.08 ± 4.65	0.19 ± 4.78	Pituitary	0.99 ± 10.46	1.26 ± 9.04
BrainStem	-0.13 ± 4.84	0.20 ± 5.00	SpinalCord PRV	0.34 ± 4.54	0.61 ± 4.45
Larynx	0.59 ± 3.09	0.73 ± 2.98	SpinalCord	0.34 ± 4.98	0.62 ± 4.96
Lens L	0.81 ± 3.77	1.05 ± 4.45	TMJ L	1.25 ± 4.35	1.01 ± 4.72
Lens R	1.21 ± 4.44	1.11 ± 4.04	TMJ R	1.85 ± 5.15	2.06 ± 5.39
Mandible L	0.76 ± 2.97	0.59 ± 2.92	Temporal Lobe L	0.15 ± 4.15	0.00 ± 4.16
Mandible R	0.66 ± 3.33	0.49 ± 3.40	Temporal Lobe R	0.09 ± 3.94	0.18 ± 3.85
Optic Chiasm	-0.23 ± 9.98	0.27 ± 8.95	Thyroid Gland	1.07 ± 5.86	1.70 ± 5.38
Optic Nerve L	0.70 ± 8.68	0.15 ± 8.80	Trachea	0.79 ± 5.94	0.81 ± 5.67
Optic Nerve R	0.66 ± 8.11	0.62 ± 7.47	Parotid R	1.17 ± 4.49	0.93 ± 4.19
Parotid L	0.60 ± 3.49	0.70 ± 3.43	PTV	0.30 ± 2.76	0.37 ± 2.79
Boost	-0.31 ± 0.56	-0.24 ± 0.57			

**Figure 4:**
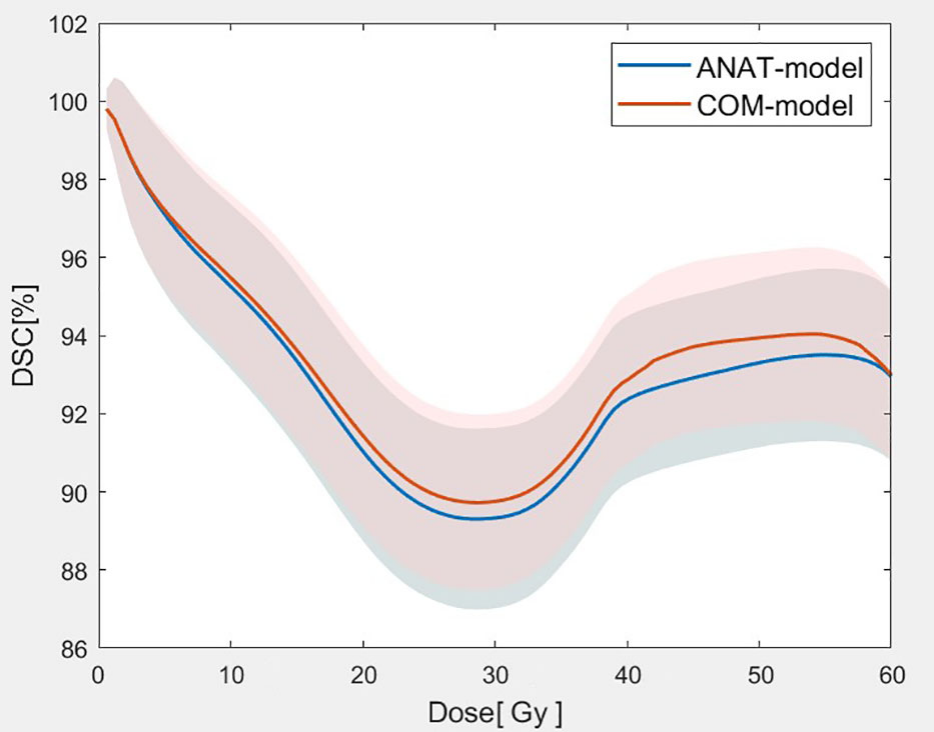
Comparison of the mean DSC values with of the isodose volumes between the two models (The solid lines represent the mean value of DSC for 2 models at each dose point and the corresponding shaded areas represent the values of standard deviation.).

As **Figure 4** shows, the mean DSC values of the isodose volumes within the range of prescription (1 Gy - 60 Gy) dose were all better for the COM model than for the ANAT model. **Table 3** shows that, the mean DSC deviations of isodose between the two models from 10 Gy to 55 Gy ranged from 0.22% to 0.80% were with highly statistical significance (p<0.001). For the DSC at 5 Gy isodose, the deviations were also statistically significant (*p* = 0.01). The lowest DSC values for the ANAT model and COM model were 89.30% and 89.72% respectively, when the isodose was approximately equal to 30 Gy.

**Table 3:** Statistical comparison of the mean DSC values of the isodose volumes between the two models.

Isodose (Gy)	ΔDSC (%)	P value	Isodose (Gy)	ΔDSC (%)	P value
5	0.09 ± 0.36	0.01	35	0.43 ± 0.50	<0.001
10	0.22 ± 0.43	<0.001	40	0.51 ± 0.42	<0.001
15	0.28 ± 0.52	<0.001	45	0.80 ± 0.39	<0.001
20	0.40 ± 0.64	<0.001	50	0.65 ± 0.38	<0.001
25	0.42 ± 0.62	<0.001	55	0.50 ± 0.34	<0.001
30	0.42 ± 0.55	<0.001	60	0.04 ± 0.42	0.293

**Figure 5:**
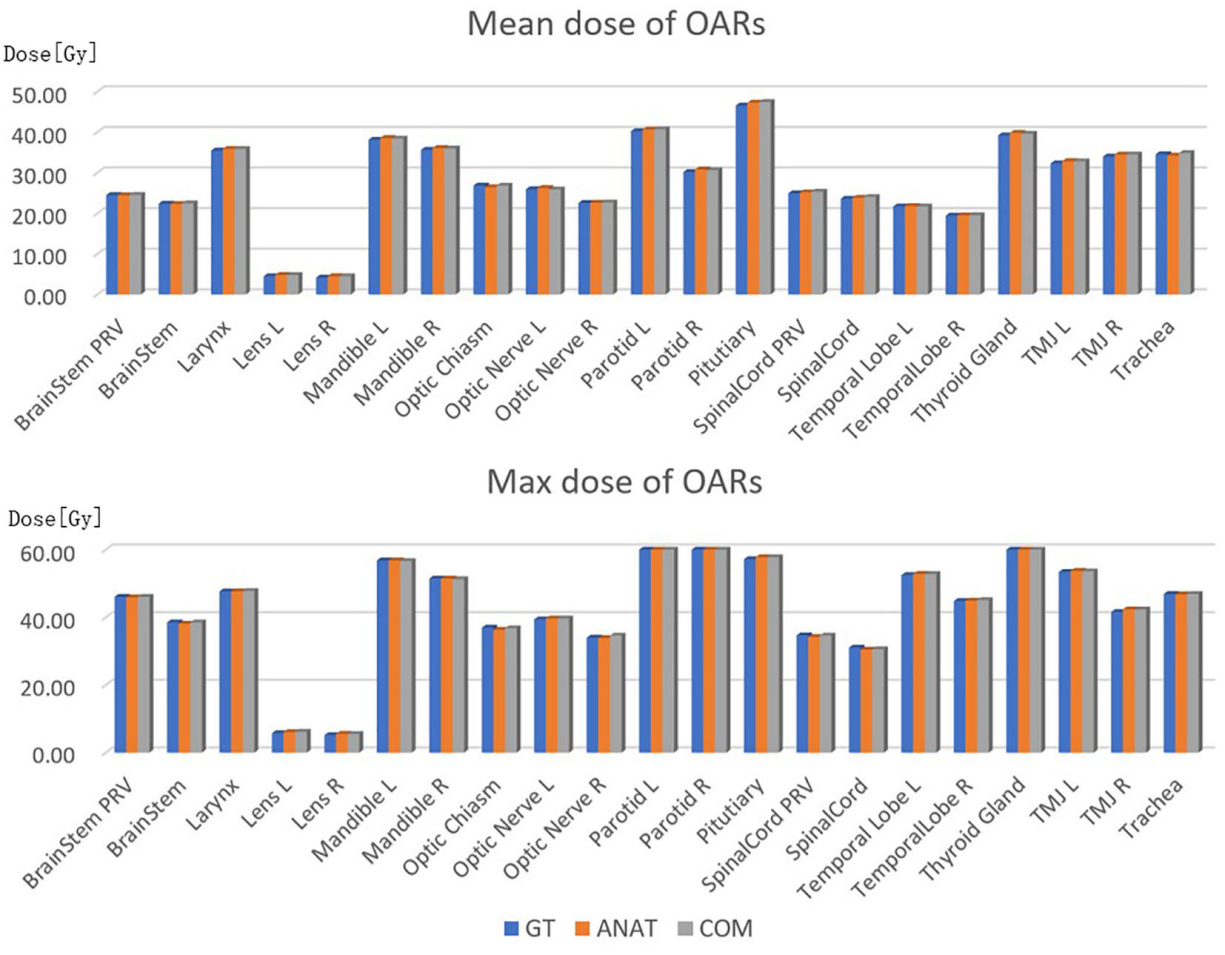
Comparison of the dosimetric parameters between the two models.

As shown in **Figure 5**, no significant differences between the dosimetric parameters predicted by the COM model and the corresponding GT value for all OARs were found (*p >0.05*). For most OARs, the dosimetric parameters predicted by the ANAT model were also quite close to the GT values, with no significant differences. Only for the right parotid was the average mean dose predicted by the ANAT model (30.89 ± 4.59 Gy), which was significantly higher than the GT value (30.15 ± 3.17 Gy). However, the absolute difference was only 0.74 Gy.

## 4 Discussion

The novelty and the significant aspect of this study with two main contributions: one is that we developed DPTV maps and combined them with direct anatomical information (including structure maps and CT) as input to train a COM deep-learning model for dose-prediction; the other is we proved the COM model could improve the accuracy of dose-prediction with highly statistical significance. The DPTV maps provided the distance relationship between each voxel in normal structures and target volumes, which might be an important complement to anatomical information for deep learning model training. Our 10-fold cross validation results demonstrate the accuracy improvement in the body dose prediction using the COM model with statistically highly significant differences, including MAE of body and DSC of most isodose volumes. The results were encouraging that the proposed COM model incorporating the distance information could achieve better dose-prediction results for whole-dose map prediction and for some OARs, which means that the DPTV maps were able to extract effective information not only for OARs but also for all voxels of body.

The DSC results of whole isodose volumes were significantly better for the COM model than for the anatomical input results, especially in the range from 10 Gy–55 Gy (*p<*0.001). For the MAE comparison of whole normal tissue and the body, the COM model also significantly outperformed the ANAT model. This finding was mainly due to the fact that the received dose of each voxel in normal tissue is related to its geometric proximity to the target. The voxels of normal tissue that were proximal to the target were likely to receive a higher dose than the dose received by distal voxels. Generating DPTV maps set as the input of one channel in the CNN model training is a simple and effective method to obtain objectively patient-specific 3D spatial information.

A high-quality optimized plan requires not only setting appropriate constraints of targets and OARs, but also considering the dose modulation by virtual volumes (VVs) in normal tissue. The VVs are designed to find the best compromise between adequate PTV coverage and satisfactory conformity to protect normal tissue as much as possible (26). For example, in inverse planning, planners create different distances of annulus -shape structures from around a target and set a series of dose constraints to make the dose conform to the target volume. The proposed COM model showed better performances in DSC and MAE of the whole normal tissue which may benefit more accurate prediction of VVs for KBRT.

Our results reveal that for most OARs, the COM model gave performance equivalent to that of the ANAT model, including voxel-based comparison and dosimetric comparison. This was mainly because the direct anatomical maps, including structure maps and CT, were set as input for training both models. The direct anatomical maps mainly focused on the shape and gray level of OARs and targets. The parameter of DPTV was one of the essential features that has been widely used for the task of dose-prediction using a convention-matching learning algorithm (11, 27). A DPTV map can extract the distance features of the body, which could have a role complementary to direct anatomical maps for obtaining high-precision dose map prediction using deep-learning.

Some researchers have proposed to incorporate extra features as inputs to improve the deep learning model of dose prediction, for example, dosimetric features. Ma et.al (21) proposed the use of contours-based features together with a PTV-only plan as the input, which was found to predict dose distribution results far more accurately compared with only contour-based features. The PTV-only plan has proved that it could seek the best PTV coverage and sacrifice sparing OARs by ignoring all OARs constraints in the deep learning model. In the future, a combination of anatomical, geometric, and dosimetric features should be carefully tested to train the deep learning model.

There were several study limitations that should be considered. First, we only used two-dimension Resnet to train the model. Another architecture should also be tested and evaluated in the future, including the 3D deep-learning method with inputs of geometric and anatomical information. Second, we only imported the NPC dataset with two-level prediction to train our dose-prediction model, so a more general prediction model should be studied in the future.

## 5 Conclusion

A deep learning model combining distance information and anatomical information was built, and a comparison showed that it outperformed a deep learning model using anatomical information only with statistically highly significant differences. This result was obtained because the distance information is also closely related to the dose distribution. The studied network model with distance and anatomical information provides a new way to obtain high-precision dose-prediction and thus has the potential to improve automated radiotherapy planning.

## Data availability statement

The original contributions presented in the study are included in the article/Supplementary Material. Further inquiries can be directed to the corresponding author.

## Ethics statement

The studies involving human participants were reviewed and approved by the Independent Ethics Committee of Cancer Hospital, Chinese Academy of Medical Sciences. Written informed consent for participation was not required for this study in accordance with the national legislation and the institutional requirements.

## Author contributions

Concept and design of study, XC, KM, and JD. Data collection and analysis, XC, JZ, and BY. Drafting the manuscript, XC. Revising the manuscript critically for important intellectual content, XC, JZ, BY, DC, KM, and JD. All authors contributed to the article and approved the submitted version.
